# Hydrogen Sulfide Alleviates Waterlogging-Induced Damage in Peach Seedlings via Enhancing Antioxidative System and Inhibiting Ethylene Synthesis

**DOI:** 10.3389/fpls.2020.00696

**Published:** 2020-05-29

**Authors:** Yuansong Xiao, Xuelian Wu, Maoxiang Sun, Futian Peng

**Affiliations:** State Key Laboratory of Crop Biology, College of Horticulture Science and Engineering, Shandong Agricultural University, Tai’an, China

**Keywords:** hydrogen sulfide, peach seedlings, antioxidative system, ethylene, waterlogging stress

## Abstract

Peach (*Prunus persica* L. Batsch) is a shallow root fruit tree with poor waterlogging tolerance. Hydrogen sulfide (H_2_S) is a signal molecule which regulates the adaptation of plants to adverse environments. Nevertheless, the effects of exogenous applications of H_2_S in fruit tree species especially in peach trees under waterlogging stress have been scarcely researched. Thus, the goal of this research was to investigate the alleviating effect of exogenous H_2_S on peach seedlings under waterlogging stress. In the present study, we found that the effect of exogenous H_2_S depended on the concentration and 0.2 mM sodium hydrosulfide (NaHS) showed the best remission effect on peach seedlings under waterlogging stress. Waterlogging significantly reduced the stomatal opening, net photosynthetic rate, and Fv/Fm of peach seedlings. The results of histochemical staining and physiological and biochemical tests showed that waterlogging stress increased the number of cell deaths and amounts of reactive oxygen species (ROS) accumulated in leaves, increased the number of root cell deaths, significantly increased the electrolyte permeability, O_2_.^–^ production rate, H_2_O_2_ content and ethylene synthesis rate of roots, and significantly reduced root activity. With prolonged stress, antioxidative enzyme activity increased initially and then decreased. Under waterlogging stress, application of 0.2 mM NaHS increased the number of stomatal openings, improved the chlorophyll content, and photosynthetic capacity of peach seedlings. Exogenous H_2_S enhanced antioxidative system and significantly alleviate cell death of roots and leaves of peach seedlings caused by waterlogging stress through reducing ROS accumulation in roots and leaves. H_2_S can improve the activity and proline content of roots, reduce oxidative damage, alleviated lipid peroxidation, and inhibit ethylene synthesis. The H_2_S scavenger hypotaurine partially eliminated the effect of exogenous H_2_S on alleviating waterlogging stress of peach seedlings. Collectively, our results provide an insight into the protective role of H_2_S in waterlogging-stressed peach seedlings and suggest H_2_S as a potential candidate in reducing waterlogging-induced damage in peach seedlings.

## Introduction

Peach (*Prunus persica* L. Batsch) is a shallow root fruit tree with high respiratory intensity, high oxygen demand, and poor waterlogging tolerance. In the cultivation of peach trees, the orchard often becomes waterlogged due to a large amount of rainfall, improper irrigation or poor drainage. Under the conditions of submergence, the air in the soil is forced to discharge, thus causing oxygen deficiency in the soil (Vartapetian and Jackson, 1997; Jackson and Armstrong, 1999) which will affect the growth and development of peach plants and thus affect the trees’ fruit yield and quality, and even lead to tree death. With global climate change and ecosystem destruction, rainstorm and flood disasters have occurred more frequently in some areas, combined with improper irrigation measures and poor soil drainage, resulting in plants suffering waterlogging damage (Jürgen and Heinz, 2014). In the peach-producing regions of southern China, the number of precipitations during the peach tree growth period is high and the rainfall time is concentrated. It is often accompanied by waterlogging, especially in the rainy season in the lower reaches of the Yangtze River. In addition, in some low-lying, poorly drained peach production areas, waterlogging also occur frequently. Waterlogging disasters have thus become an obstacle to the cultivation of peach trees. Therefore, how to reduce the harm of waterlogging disasters to peach trees through technical measures has important theoretical and practical value.

Waterlogging stress can impair growth and entry into production of young fruit trees, and reduce the growth, yield and fruit quality of mature trees (Penella et al., 2017). Under hypoxic stress, aerobic respiration is inhibited in root and a large amount of toxic substances, such as ethanol and lactate, are accumulated in the root system (Guo et al., 1998; Morard et al., 2000). The damage to plants from long-term waterlogging is mainly caused by secondary stresses, such as hypoxia caused by excessive water content, blocked electrolyte transmission in plant cells, and accumulation of reactive oxygen species (ROS), that affect the cell membrane structure and function (Vantoai and Bolles, 1991; Irfan et al., 2010). If water stress is prolonged and/or severe, part of the energy supplied by incident photons maybe redirected into processes that favor the generation of ROS such as hydrogen peroxide (H_2_O_2_) and superoxide anion (O_2_.^–^) leading to oxidative damage to plant tissues (Jiang and Zhang, 2002) and affect the cellular homeostasis and other negative impacts of water logging inside the plants. Nevertheless, plants can activate ROS-scavenging enzymes such as superoxide dismutase (SOD), catalase (CAT) to reduce oxidative damage (Gill and Tuteja, 2010; Tattini et al., 2015).

Hydrogen sulfide (H_2_S) is the third gas signal molecule discovered after nitric oxide (NO) and carbon monoxide (CO) in animals and plants (Hancock and Whiteman, 2016; Fu et al., 2018). It can be used as a signal molecule to regulate plant growth and development and stress responses (He et al., 2019a). H_2_S is part of a suite of small reactive molecules which are known to be involved in cell signaling events in plants (Hancock, 2019). The latest findings indicate that H_2_S may play a role in stomatal signaling (Garcia Mata and Lamattina, 2010; Lisjak et al., 2010) and may promote the synthesis of chloroplasts (Chen et al., 2011). H_2_S participates in seed germination, stomatal movement, root growth and development (Lin et al., 2012; Fang et al., 2014). H_2_S can enhance photosynthesis of plant leaves, enhances stress tolerance, and affects other plant physiological processes (Zhang et al., 2009; Garcia Mata and Lamattina, 2010; Lisjak et al., 2010; Chen et al., 2018). H_2_S can also alleviate the damage caused by multiple abiotic stresses, such as osmotic pressure, heavy metal ions, and drought (Qiao et al., 2015; Chen et al., 2016; Lv et al., 2017; Zhu et al., 2018; Kolupaev et al., 2019; He et al., 2019b; Kaya et al., 2020). Li et al. (2013a) reported that sodium hydrosulfide-improved heat tolerance in maize and involvement of proline.

It has been reported to enhance the plant’s resistance under waterlogging stress by enhancing the antioxidant enzyme system in tomato (Murshed et al., 2013), and in fruit trees such as mandarin (Sarkar et al., 2016). Research has shown that H_2_S can improve maize seed germination and seedling growth under high temperature by inducing antioxidant system and osmolyte biosynthesis (Zhou et al., 2018) and alleviate the damage to pea plants caused by waterlogging stress (Cheng et al., 2013). Application of H_2_S decreased the production of ROS in the plant leaves and roots by increasing antioxidant activities and had a protective role on plant growth, photosynthetic parameters, elements uptake under stress conditions (Ali et al., 2014a,b,c). H_2_S can lead to changes in the activity of antioxidants under environmental stresses (Hancock, 2019). Reports showed that H_2_S pretreatment can increase the activities of peroxidase (POD), SOD, CAT, and ascorbate peroxidase (APX) and reduce ROS content when plants respond to waterlogging stress and cadmium toxicity stress (Cheng et al., 2013; Sun et al., 2013; Shi et al., 2015). Cheng et al. (2013) indicated that H_2_S pretreatment inhibited ethylene biosynthesis and alleviates hypoxia-induced root tip death in pea seedlings. In addition, it is found that H_2_S is also involved in the Eth-induced stomatal closure process (Liu et al., 2011). These show that exogenous H_2_S has positively impact physiological parameters in herbaceous plants under waterlogging stress. Nevertheless, the effects of exogenous applications of H_2_S in fruit tree species especially in peach trees under waterlogging stress have been scarcely researched.

Thus, the goal of this research was to investigate the alleviating effect of exogenous H_2_S on peach seedlings under waterlogging stress. In this study, peach seedlings were used as the test material, and the H_2_S releasing agent sodium hydrosulfide (NaHS) and its scavenger hypotaurine (HT) were applied under the conditions of waterlogging. The injury to peach plants under waterlogging stress could be alleviated by exogenous H_2_S, as revealed via histochemical staining and physiological and biochemical methods. We investigated the effect of exogenous H_2_S at various concentrations on peach seedlings under waterlogging stress, and we further characterized the effect of 0.2 mM NaHS, which showed the best mitigation effect. And the 0.2 mM NaHS was used to further investigate the mechanism of exogenous H_2_S alleviating waterlogging stress. The purpose of this study was to explore whether H_2_S could alleviate the damage of waterlogging stress on peach trees by regulating antioxidative system and ethylene synthesis in roots, and to provide new research ideas and a scientific basis for reducing or overcoming waterlogging damage to peach trees.

## Materials and Methods

### Plant Material and Treatments

Peach seeds with the same size treated by stratification were seeded in seedling trays. When the seedlings had grown to have 5 or 6 true leaves, the plants with the same growth level and without pest, or disease damage were selected and planted in plastic basins 12 cm in diameter and 13 cm in height. The cultivation medium was quartz sand, and the surface of the quartz sand was 10 cm away from the upper edge of the basin. One plant was planted in each basin, and plants were maintained with routine management. The potted seedlings were placed in a water storage tank with water for the waterlogging treatments. The dimensions of the water storage tank were length × width × height = 70 cm × 35 cm × 17 cm.

Firstly, we investigated the effects of exogenous H_2_S with various concentrations on net photosynthetic rate (Pn) and chlorophyll content of peach seedlings after 72 h waterlogging treatment. The treatments were as follows: non waterlogging (Control), waterlogging (WL), waterlogging + 0.02 mM NaHS (WL + 0.02 NaHS), waterlogging + 0.05 mM NaHS (WL + 0.05 NaHS), waterlogging + 0.1 mM NaHS (WL + 0.1 NaHS), waterlogging + 0.2 mM NaHS (WL + 0.2 NaHS), and waterlogging + 0.3 m M NaHS (WL + 0.3 NaHS).

We further characterized the alleviation of waterlogging stress by 0.2 mM NaHS, which had shown the best effect on waterlogging stress mitigation. The treatments were as follows: non waterlogging (Control), WL, waterlogging + 0.2 mM NaHS (WL + 0.2 NaHS), waterlogging + 0.2 mM NaHS + 0.1 mM H_2_S scavenger HT (WL + 0.2 NaHS + 0.1HT), and waterlogging + 0.1 mM H_2_S scavenger HT (WL + 0.1 HT). For the waterlogging treatments, the liquid level in the water storage tank was always 1 cm higher than that in the plastic basin, and 1 in 20 basins were treated. Plant samples were collected at 0, 24, 48, and 72 h after treatment, and the related indexes were determined. The experiment was repeated three times, and the average values of the results were used in the analysis.

### Measurement of Photosynthetic Characteristics of Leaves

The photosynthetic characteristics of leaves were measured at 0, 24, 48, and 72 h after treatment using a ciras-3 portable photosynthetic instrument (PPSystems, United Kingdom). Net Pn of leaves was measured at 9:00–11:00, and Fv/Fm was measured with a fluorometer (Handy PEA, Hansatech, United Kingdom).

### Determination of Chlorophyll Content

The content of chlorophyll was measured 72 h after treatment. Chlorophyll a, chlorophyll b, and carotenoids were extracted with 95% ethanol, and the absorbances of chlorophyll a, chlorophyll b, and carotenoids at 663.3, 646.8, and 470 nm were measured with a spectrophotometer (Arnon, 1949).

### Histochemical Staining of O_2_^–^ and H_2_O_2_

The leaves were stained via histochemistry 72 h after treatment. The 3′, 3′-diaminobenzidine (DAB) and nitroblue tetrazolium (NBT) staining were conducted as described by Hu et al. (2016) with modification. The color development of superoxide anion and hydrogen peroxide was observed by histochemical staining with NBT and DAB, respectively. The leaves of peach seedlings with different treatments were put into NBT (pH = 7.8) and DAB (pH = 5.0) solutions containing 0.5 mg/ml. In the dark, the leaves were stained at room temperature for 4 h and then transferred into 75% ethanol. Then, O_2_^–^ and H_2_O_2_ content in the leaves were observed by taking photos. The intensity of the fluorescent signals was quantified using the ImageJ software.

### Measurements of Stomatal Density and Size

Stomatal density and size was measured 72 h after treatment. Three leaves of the same developmental stage were selected to determine the size and density of the stomata. To do so, we applied transparent acrylic nail polish to the epidermis of peach leaves. Once the nail polish had dried, it was peeled off using forceps. The solid polish was then placed on a microscope slide and observed using a Fluorescence Microscope under 400 × magnification (AXI0, Carl Zeiss, Germany). We selected three areas of 3.2 mm^2^ each slide randomly and captured an image. We counted the number of stomata and determined their length and width using ImageJ version 1.48 (National Institutes of Health, Bethesda, MD, United States).

### Determination of H_2_S Content in Roots

The content of H_2_S was determined with reference to the methylene blue method of Sekiya et al. (1982) with slight modification. Samples of 0.1 *g* peach root, with 0.9 mL 20 mmol L^–1^ Tris–Hydrogen chloride buffer (pH = 8.0), were ground to a homogenate, centrifuged, and the supernatant was placed into a 500-μl test tube, after which 2 ml of homogenate and a certain amount of zinc acetate were added. After that, 100 μL 30 mmol L^–1^ Ferric chloride solution and 100 μL 20 mmol L^–1^ *N*, *N*-dimethyl-p-phenylenediamine were added into the test tube. The tubes were stoppered and held at room temperature for 30 min, and then the absorbance was measured at 670 nm.

### Determination of Protective Enzyme Activity

According to the method of Chen and Patterson (1988), the activity of SOD was determined by inhibiting the photoreduction of NBT by 50% per minute, and the result was expressed as U g^–1^ protein; the activity of POD was determined by the method of Omran (1980) and was expressed as the amount of enzyme needed to reduce 0.01 of the value per minute. The activity of CAT was determined by the method of Kar and Mishra (1976). The amount of enzyme needed to reduce 0.01 of the value per minute was 1 active unit (U), and the activity of POD and CAT was expressed as μmol mg^–1^ min^–1^ protein.

### Measurement of Electrolyte Permeability of Roots

The electrolyte permeability was measured after 72 h of treatment according to the method of Fan et al. (1997). Briefly, the conductivity of root (0.5 *g*) was measured in deionized water at room temperature for 2 h. Subsequently, the solution was heated in boiling water for 10 min and cooled to room temperature to determine total conductivity. The conductivity was expressed as the percentage of the initial conductivity versus the total conductivity.

### Determination of Proline Content in Roots

The proline concentration was measured after 72 h of treatment according to the method of Bates et al. (1973). Chopped roots (0.5 *g*) were placed into large test tubes; 5 ml of 3% sulfosalicylic acid solution was added; the nozzle was covered with a glass ball plug, and the extraction was performed in a boiling water bath for 10 min. The test tube was cooled to room temperature, and 2 ml of supernatant was added to 2 ml of glacial acetic acid, and 3 ml of color developing solution. The solution was heated in a boiling water bath for 40 min, cooled to room temperature, and 5 ml of toluene added. The solution was shaken vigorously to extract the red substance, and then left standing to wait for layering. The toluene layer was drawn off to measure the absorbance at 520 nm.

### Determination of Reactive Oxygen Species Levels

Determination of O_2_^–^ production rate was performed according to Elstner and Heupel (1976), where 1 *g* of fresh peach root was ground with 3 ml phosphate buffer (pH = 7.8), then centrifuged at 4,000 *g* for 15 min. Then, 0.5 ml of the supernatant liquid was added to 1 ml hydroxylamine and incubated at 25^°^C for 1 h. Then, 1 ml reaction liquid was mixed with 17 mM p-aminobenzene sulfonic acid and 7 mM a-naphthylamine solution before additional 20 min incubation at 25^°^C. The reaction mixture was used to measure the absorbance at 530 nm, and the O_2_^–^ product activity rate was calculated by a linear calibration curve of Sodium nitrite (NaNO_2_).

Hydrogen peroxide (H_2_O_2__)_ content was assayed according to Patterson et al. (1984) based on appropriate improvements. Approximately 0.1 *g* of root was weighed, quickly cut into pieces, placed into a centrifuge tube, and frozen with liquid nitrogen. The tube was centrifuged at 60 rpm for 150 s, shaken, and then repeated once; added 1.5 ml 0.1% Trichloroacetic Acid (TCA), quickly mixed, and the tube put on ice. The tube was centrifuged at 12000 rpm for 15 min at 4^°^C; 0.5 ml of the supernatant was taken into a clean 2-ml centrifuge tube; 0.5 ml Phosphate buffer saline (PBS) and 1 ml Potassium iodide (KI) were added; sufficient vibration was carried out, and the temperature was kept at 28^°^C for 1 h, after which the absorption was read at 390 nm.

### Determination of Root Activity

Root activity was measured after 72 h of treatment using the triphenyltetrazolium chloride method (TTC method) following Zou (2000). Approximately 0.5 *g* of root was placed into 10 ml of the equivalent mixture of 0.4% TTC solution and phosphate buffer (pH 7.0) and insulated in the dark for 1–3 h at 37^°^C. Then, 2 ml of 1 molar sulfuric acid was added to stop the reaction (A blank experiment was done simultaneously by first adding sulfuric acid and root samples, then adding other reagents after 10 min). The root was removed and dried, then ground with 3–4 ml ethyl acetate and a small amount of quartz sand together in a mortar to extract the TTC. The red extract was transferred to a test tube, and the residue was washed twice with a small amount of ethyl acetate. Finally, 10 ml ethyl acetate was added, and the colorimetric assay was performed with a spectrophotometer at a wavelength of 485 nm.

### Determination of Cell Death

The leaves and roots of peach seedlings were stained with Evans Blue after 72 h of treatment, and photos were taken under a stereomicroscope (Steffens and Sauter, 2009). Leaves or roots were soaked in Evans Blue solution 0.25% (W/V) for 24 h. The leaves and roots were removed and cleaned with pure water and placed into a solution of anhydrous ethanol: glycerin (4:1) and boiled until the bottom color of the leaves turned white. The leaves and roots were then photographed.

### Determination of Ethylene Production Rate

Peach root (0.3 *g*) was placed in a 5-ml penicillin bottle and kept at room temperature for 2 h. Then gas chromatography (VARIANCP-3380, equipped with a PONA chromatographic column) was used to analyze the ethylene content in the root tip cells, i.e., standard ethylene injection was used first; the peak time of ethylene was accurately recorded, and the standard curve was made; then the gas in the bottle was aspirated for 600 μl injection, and the ethylene content measured by calculating the peak area of ethylene and referring to the standard curve.

### Data Analyses

DPS software was used to collect data, and analysis of differences was processed using Duncan’s new multiple range method. The differences among means and correlation coefficients were considered to be significant when *p* < 0.05.

## Results

### Effect of Exogenous H_2_S With Different Concentrations on Net Photosynthetic Rate and Chlorophyll Content of Peach Seedlings of Waterlogging Treatment

Chlorophyll is the material basis of photosynthesis in plant leaves, and its content can reflect the growth status and photosynthetic capacity of leaves (Nieva et al., 2005). It has been found that when the growth of plants is inhibited by waterlogging, the chlorophyll content is reduced (Edwards et al., 2003), and the net photosynthetic rate at the initial stage is sharply reduced (Ahmed et al., 2002).

To determine the optimal concentration of exogenous H_2_S (NaHS treatment), we choose 0, 0.02, 0.05, 0.1, 0.2, and 0.3 mM NaHS treatment under the condition of waterlogging. Exogenous H_2_S had a dose-dependent effect on alleviating chlorophyll degradation and photosynthesis inhibition of peach seedlings under waterlogging stress. As shown in Table 1, after 72 h of treatment, the net photosynthetic rate of peach seedlings leaves decreased significantly under waterlogging stress. Compared with the control, the net photosynthetic rate decreased by 70.0% under 0 mM NaHS treatment, and 0.02–0.3 mM NaHS treatment could alleviate the inhibition of salt water stress on the photosynthesis of peach seedlings leaves to a certain extent. Compared with the control, the chlorophyll a, chlorophyll b, and chlorophyll a + b in the leaves of peach seedlings were significantly reduced under waterlogging stress. Exogenous H_2_S treatment could effectively alleviate this trend, and 0.2 mm NaHS treatment had the best effect. The results showed that exogenous H_2_S could effectively alleviate the degradation of chlorophyll and the inhibition of photosynthesis in the leaves of peach seedlings under waterlogging stress. The best concentration of NaHS was 0.2 mM.

**TABLE 1:** Effect of exogenous H_2_S with different concentrations on net photosynthetic rate and chlorophyll content in peach seedlings leaves under waterlogging stress.

Treatments	Control	Waterlogging + NaHS					
		0 mM	0.02 mM	0.05 mM	0.1 mM	0.2 mM	0.3 mM
Pn (μM CO_2_ m^–2^ s^–1^)	13.67 ± 0.551^a^	4.10 ± 0.400^f^	4.77 ± 0.513^ef^	5.50 ± 0.656^e^	7.53 ± 0.737^d^	9.80 ± 0.361^b^	8.63 ± 0.451^c^
Chlorophyll a (mg g^–1^ FW)	0.65 ± 0.001^a^	0.58 ± 0.004^f^	0.59 ± 0.005^ef^	0.60 ± 0.002^e^	0.62 ± 0.006^d^	0.64 ± 0.001^b^	0.63 ± 0.005^c^
Chlorophyll b (mg g^–1^ FW)	0.20 ± 0.009^a^	0.15 ± 0.007^e^	0.16 ± 0.003^d^	0.16 ± 0.001^cd^	0.17 ± 0.004^c^	0.18 ± 0.004^b^	0.17 ± 0.002^bc^
Chlorophyll a+b (mg g^–1^ FW)	0.85 ± 0.010^a^	0.73 ± 0.010^e^	0.75 ± 0.008^d^	0.76 ± 0.003^d^	0.78 ± 0.010^c^	0.81 ± 0.004^b^	0.80 ± 0.006^c^

*Pn, Net photosynthetic rate (μM CO_2_ m^–2^ s^–1^); Significant differences (*P* < 0.05) between treatments were indicated by different small letters. Values represent the averages of three biological replicates ± SD.*

### Effect of Exogenous H_2_S on Stomatal Characteristics of Peach Seedlings Leaves Under Waterlogging Stress

**FIGURE 1:**
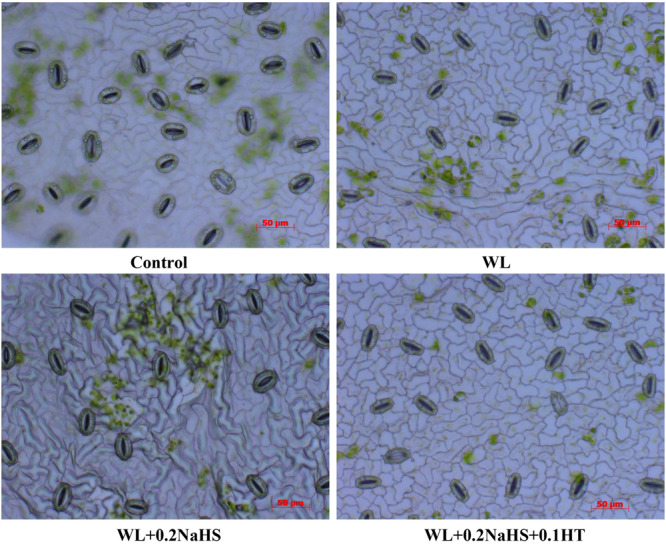
Effects of exogenous H_2_S on stomatal characteristics in peach seedlings leaves under waterlogging stress.

Stomata are the “windows” for gas exchange between plants and the outside world. Stomatal conductance affects leaf photosynthetic rate, intercellular carbon dioxide concentration, and transpiration rate, all of which are sensitive to stress. Compared with the non-waterlogging treatment, the stomatal openings of the flooded leaves were smaller, and the stomatal openings of the flooded leaves were significantly smaller than those of the control. Applying exogenous H_2_S could reduce the stomatal openings of the leaves (Figure 1); compared with WL treatment, WL + 0.2 NaHS treatment significantly increased the average stomatal length and stomatal width, by 11.36% and 27.12%, respectively, (Table 2). However, compared with the treatment of WL, the treatment of WL + 0.2 NaHS + 0.1 HT with exogenous H_2_S and H_2_S scavenger HT significantly increased the stomatal length and stomatal width by 5.0% and 15.25%, respectively.

**TABLE 2:** Effect of exogenous H_2_S on stomatal length and width in peach seedlings leaves under waterlogging stress.

Treatment	Control	WL	WL + 0.2NaHS	WL + 0.2NaHS + 0.1HT
Stomatal length (μm)	25.30 ± 0.907^a^	22.03 ± 0.520^d^	24.48 ± 0.639^b^	23.10 ± 0.917^c^
Stomatal width (μm)	9.30 ± 0.515^a^	5.94 ± 0.488^d^	7.46 ± 0.709^b^	6.84 ± 0.527^c^

*Significant differences (*P* < 0.05) between treatments were indicated by different small letters. Values represent the averages of three biological replicates ± SD.*

### Effect of Exogenous H_2_S on Cell Death and ROS Accumulation of Peach Seedlings Leaves Under Waterlogging Stress

**FIGURE 2:**
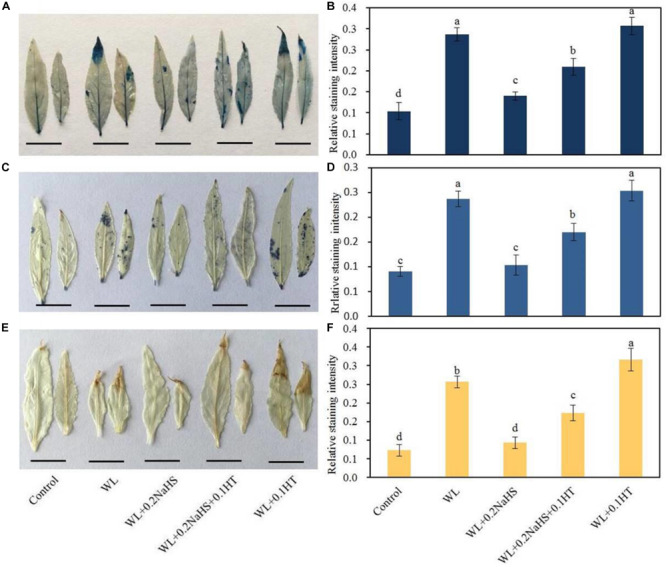
Effects of exogenous H_2_S on cell death and ROS accumulation of peach seedlings leaves under waterlogging conditions. **(A)** Staining with Evans Blue to show areas of cell death; **(B)** Evans Blue staining intensity as determined with ImageJ software. **(C)** Distribution of superoxide anion radicals (O_2_^–^) visualized by nitroblue tetrazolium (NBT), and **(D)** NBT staining intensity as determined with ImageJ software. **(E)** hydrogen peroxide (H_2_O_2_) was visualized by 3,3-diaminobenzidine (DAB) staining in peach leaves. **(F)** DAB staining intensity as determined with ImageJ software. In (B; D; F) each data point represents the mean (with SD bar) of three replicates. Values followed by different letters in the same day are significantly different at *P* < 0.05.

Waterlogging can cause oxidative damage and cell injury. Therefore, we measured the cell death and ROS accumulation of peach seedlings leaves under different treatment conditions. We used Evans Blue staining, DAB staining, and NBT staining to compare the cell death and ROS accumulation of peach seedlings leaves under different treatment conditions. It can be seen from Figures 2A,C,E that under the waterlogging stress, the color of the leaves was darker and the staining area was larger, indicating that under the waterlogging stress, the cells in the leaves of peach seedlings were damaged, the number of dead cells was increased, and the ROS production in the leaves was significantly increased. Under waterlogging stress, the stained areas of leaves treated with exogenous H_2_S were smaller, indicating that exogenous H_2_S could alleviate the damage from waterlogging stress on peach seedlings leaves, significantly reduce the amount of cell death, and significantly reduce ROS production (Figures 2B,D,F).

### Effect of Exogenous H_2_S on Photosynthetic Characteristics of Peach Seedlings Leaves Under Waterlogging Stress

**FIGURE 3:**
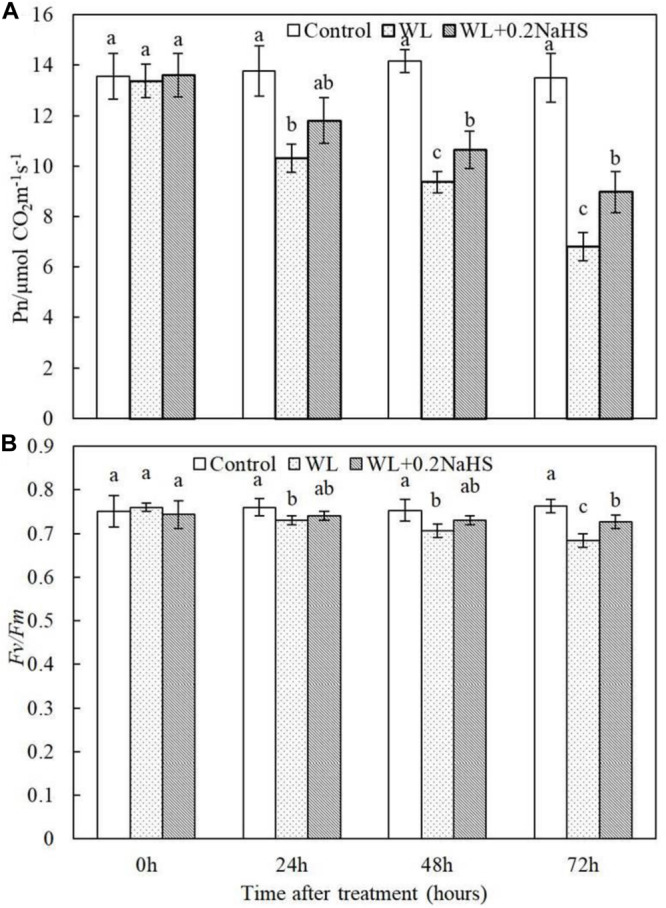
Effects of exogenous H_2_S on **(A)** photosynthetic rates and the **(B)** maximum photochemistry effciency of PS II (Fv/Fm) in the peach seedlings leaves under waterlogging conditions. Each data point represents the mean (with SD bar) of three replicates. Values followed by different letters in the same time are significantly different at *P* < 0.05.

Fv/Fm represents the maximum photochemical quantum yield under dark adaptation, and its value reflects the conversion efficiency of primary light energy in the PSII reaction center, a good indicator of light inhibition degree. PIABS is a parameter reflecting the comprehensive photosynthetic performance of plants. The fluorescence parameter Fv/Fm represents the primary light energy conversion efficiency of PS II (Demmig and Bjorkman, 1987). We measured the net photosynthetic rate and Fv/Fm of peach seedlings leaves at 0, 24, 48, and 72 h after waterlogging. It can be seen from Figure 3 that the net photosynthetic rate and Fv/Fm of peach seedlings leaves were significantly reduced by waterlogging stress, while the net photosynthetic rate and Fv/Fm of peach seedlings leaves were significantly higher under exogenous application of H_2_S than under waterlogging alone. The results showed that exogenous H_2_S could maintain relatively high net photosynthetic rate and electron efficiency.

### Effect of Exogenous H_2_S on the Content of Endogenous H_2_S in the Root System of Peach Seedlings Under Waterlogging Stress

**FIGURE 4:**
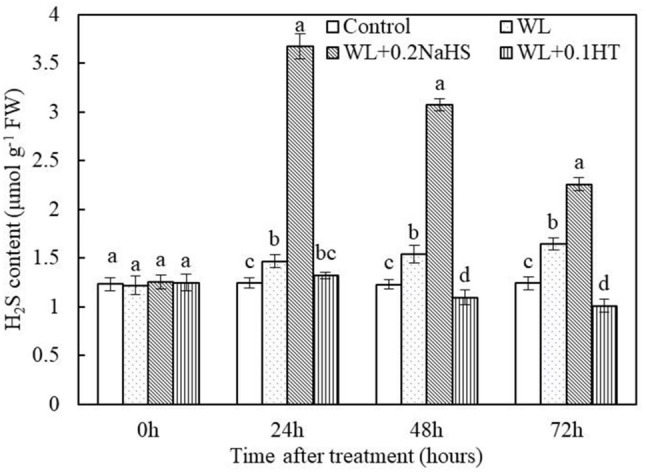
Effects of exogenous H_2_S on internal content in peach seedlings roots under waterlogging conditions. Each data point represents the mean (with SD bar) of three replicates. Values followed by different letters in the same time are significantly different at *P* < 0.05.

In order to study the effect of waterlogging stress on the content of endogenous H_2_S in peach roots, we measured the content of endogenous H_2_S in peach roots under different treatment conditions. It can be seen from Figure 4 that compared with the control, the content of endogenous H_2_S in peach root increased significantly after 24 h of waterlogging treatment. At the same time, the application of exogenous H_2_S under waterlogging stress significantly increased the content of endogenous H_2_S in peach roots; this may have been caused by the spread of exogenous H_2_S to root cells. The results showed that the content of endogenous H_2_S in root tip cells increased significantly under waterlogging stress, and the application of exogenous H_2_S could further increase the content of endogenous H_2_S in the root system.

### Exogenous H_2_S Alleviates Root System Damage of Peach Seedlings Under Waterlogging Stress

Oxygen is necessary for the respiration of the plant root system, and the root system is the first to be affected under waterlogging conditions. Root hypoxia inhibits the aerobic respiration of the root system, destroying its normal physiological functions and possibly leading to root apoptosis. The long-term inundation results in the air being discharged from the soil, resulting in an anoxic environment in the root zone; this inhibits the aerobic respiration of the crop root system and affects the root activity. When the plant tissue is injured, the structure of the membrane is damaged, increasing the root permeability. Many water-soluble substances in the cell, including electrolytes, will have different degrees of extravasation. When the plant tissue is immersed in non-ionic water, the conductivity of the water will increase due to the extravasation of electrolytes. Because the plant cell membrane is easily damaged by oxidative damage, this eventually leads to cell death.

**FIGURE 5:**
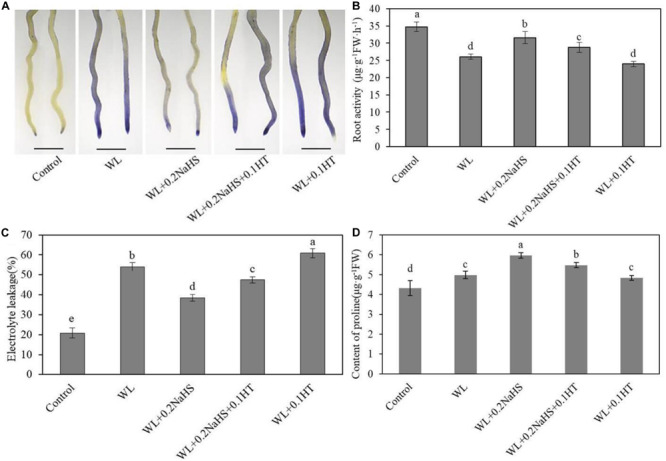
Effect of exogenous H_2_S on the membrane integrity and proline concentration in peach seedlings root under waterlogging conditions. **(A)** Staining with Evans Blue to show areas of cell deaths. In **(B–D)** each data point represents the mean (with SD bar) of three replicates. Values followed by different letters in the same time are significantly different at *P* < 0.05.

In order to study whether exogenous H_2_S can affect the injury degree and cell membrane integrity of root system cells of peach seedlings under waterlogging stress, we used the Evans Blue histochemical staining method to determine root cell death, root activity, and cell membrane permeability under different treatment conditions. As shown in Figure 5A, the color of roots under waterlogging stress was darker, while that under exogenous H_2_S treatment was lighter. In addition, under exogenous H_2_S treatment, the root activity of peach seedlings increased significantly (Figure 5B), and the electrolyte permeability of roots decreased significantly (Figure 5C).

In addition, proline is an important osmotic regulator in plant cells. Plants can resist stress by increasing proline content, and thus proline can reflect the stress resistance to a certain extent. We measured the content of proline in roots under different treatment conditions. As shown in Figure 5D, exogenous H_2_S treatment increased the proline content of the root system, thereby enhancing the resistance of the plant to waterlogging stress.

### Effects of Exogenous H_2_S on ROS Accumulation of Peach Seedlings Under Waterlogging Stress

**FIGURE 6:**
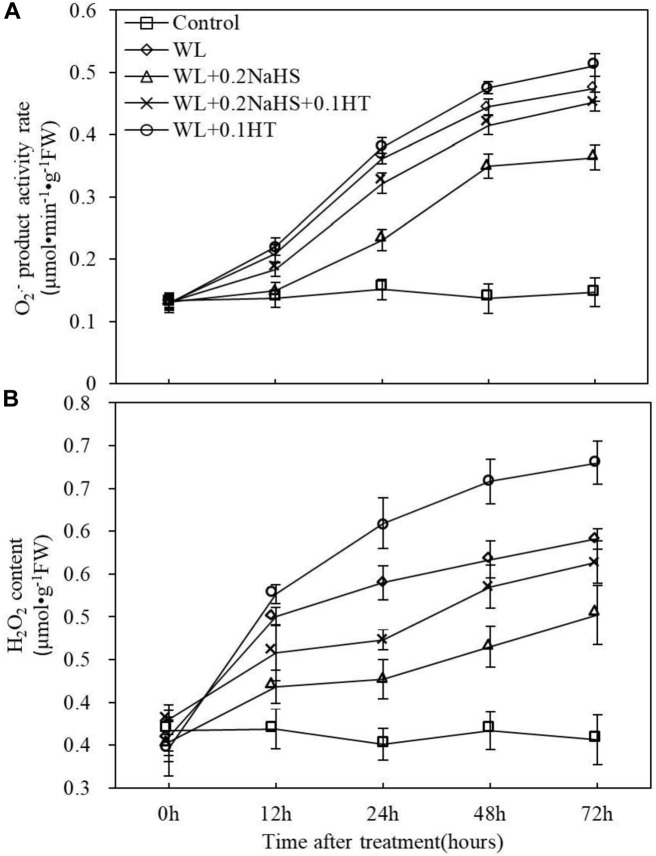
Effects of exogenous H_2_S on ROS metabolize in peach seedlings roots under waterlogging conditions. **(A)** Superoxide radical ion (O_2_^–^) product activity rate. **(B)** Hydrogen peroxide (H_2_O_2_) content. Each data point represents the mean (with SD bar) of three replicates. Values followed by different letters in the same day are significantly different at *P* < 0.05.

The accumulation of ROS in root tip cells may be the main cause of root death of peach seedlings under waterlogging stress, and exogenous H_2_S can significantly alleviate the degree of root death under waterlogging stress (Figure 5). Therefore, the rate of O_2_^–^ production and the content of H_2_O_2_ in apical cells were determined. The results showed that waterlogging stress significantly increased the O_2_^–^ production rate and H_2_O_2_ content in the root system of peach seedlings, while exogenous H_2_S could reduce the O_2_^–^ production rate and H_2_O_2_ accumulation in the root system of peach seedlings (Figure 6). However, adding the H_2_S scavenger HT eliminated/partially eliminated the effect of H_2_S. The results showed that exogenous H_2_S could reduce the accumulation of ROS in the root system of peach seedlings under waterlogging stress, thereby alleviating the oxidative damage to the roots caused by waterlogging stress.

### Exogenous H_2_S Enhances Antioxidant Capacity of Root Cells of Peach Seedlings Under Waterlogging Stress

**FIGURE 7:**
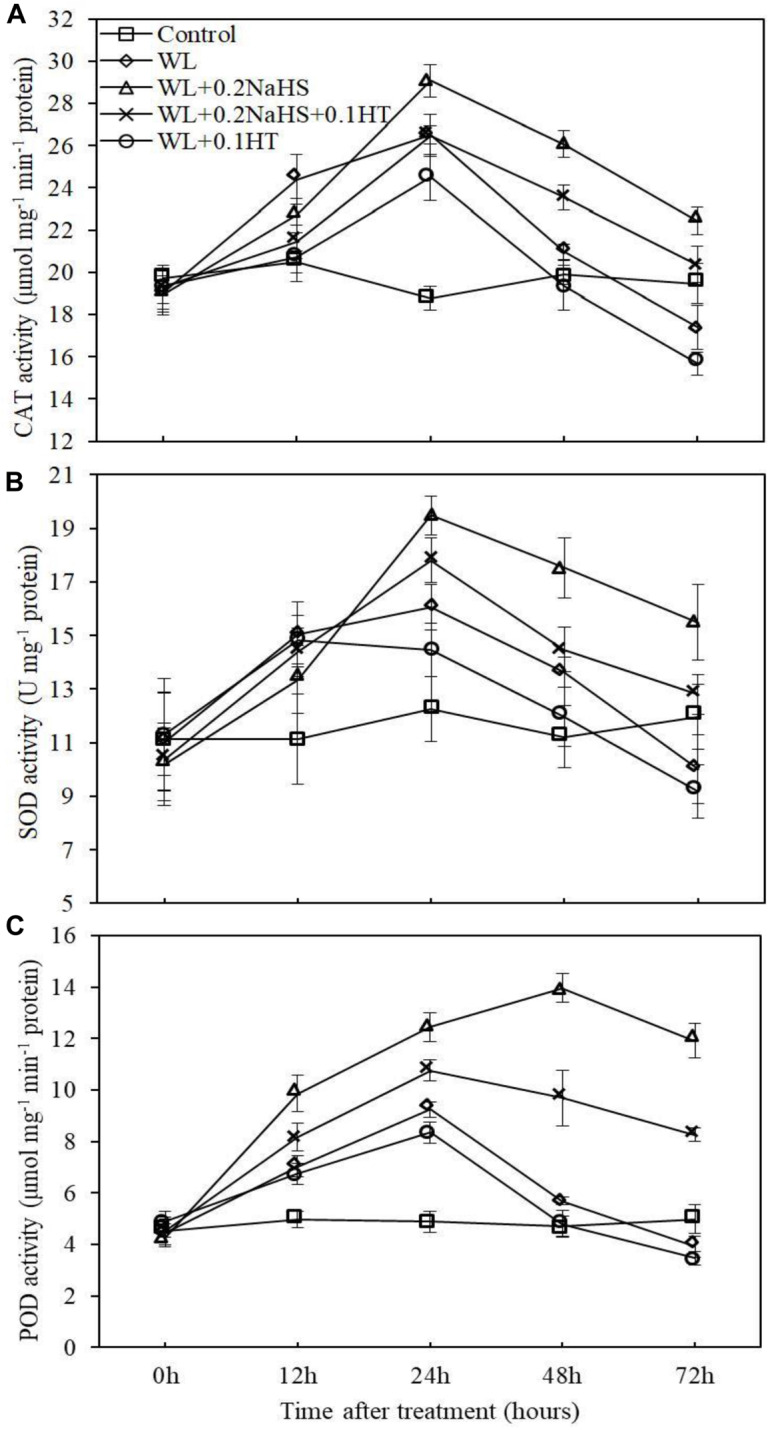
Effects of exogenous H_2_S on antioxidant enzymes activities in peach seedlings roots under waterlogging conditions. **(A)** Catalase (CAT) activity. **(B)** Superoxide dismutase (SOD) activity. **(C)** Peroxidase (POD) activity. Each data point represents the mean (with SD bar) of three replicates. Values followed by different letters in the same day are significantly different at *P* < 0.05.

Plants contain antioxidant enzymes, such as superoxide dismutase, peroxidase and catalase (CAT, SOD, and POD), that can clear away ROS and avoid cell damage (Blokhina et al., 2000; Ke et al., 2003). In order to analyze the effect of exogenous H_2_S treatment on the antioxidant system activity of peach seedlings root cells under waterlogging stress, we measured the activities of CAT, SOD, and POD in root cells. As shown in Figure 7, the activities of CAT, SOD, and POD in the root systems of peach seedlings were significantly increased after 12 h of waterlogging.

### Exogenous H_2_S Inhibits Ethylene Production in Roots of Peach Seedlings Under Waterlogging Stress

**FIGURE 8:**
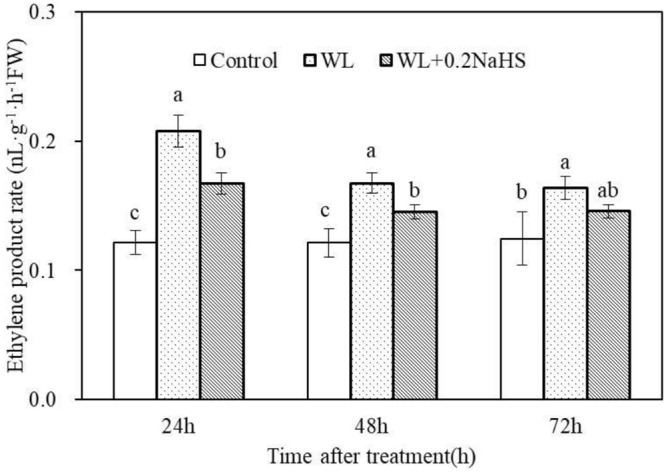
Effects of exogenous H_2_S on ethylene biosynthesis in peach seedlings roots of waterlogging conditions. Each data point represents the mean (with SD bar) of three replicates. Values followed by different letters in the same time are significantly different at *P* < 0.05.

Hypoxia stress can induce the production of plant hormone ethylene, and ethylene can regulate various response strategies of plant cells under waterlogging stress. Therefore, we measured the effect of exogenous H_2_S on the ethylene content of root systems of peach seedlings under waterlogging stress. As shown in Figure 8, waterlogging stress promoted root ethylene synthesis and increased the root ethylene synthesis rate. Exogenous H_2_S treatment significantly reduced the ethylene content in the root system of peach seedlings. The results demonstrated that exogenous H_2_S could help the plants adapt to a low oxygen environment caused by waterlogging stress by inhibiting ethylene synthesis in the root system.

## Discussion

Oxygen is necessary for the respiration of plant root systems, and the root system is the first to be affected under waterlogging conditions. Root hypoxia inhibits the aerobic respiration of the root system, destroys the normal physiological function of the root system, and even leads to root apoptosis. The long-term inundation results in the air being discharged from the soil, and the resulting anoxic environment in the root zone inhibits the aerobic respiration of the crop root system and affects the root activity. When the plant tissue is injured, the function of the root membrane is damaged or the structure is destroyed, increasing the root permeability. Many water-soluble substances in the cell, including electrolytes, will have different degrees of extravasation. When the plant tissue is immersed in non-ionic water, the conductivity of the water will increase due to the extravasation of electrolytes. The more serious the injury, the greater the degree of extravasation and the increase in conductivity (Hu et al., 2016). The proline content of plant cells increases exponentially under waterlogging stress to maintain the cell pressure and protect the membrane system from injury (Wang et al., 1998). Proline, as an osmoregulatory substance, can alleviate the damage from abiotic stress to the cell membrane to a certain extent (Wan et al., 2015; Cheng et al., 2018). The results showed that the root activity and proline content were significantly higher, the electrolyte permeability of roots was significantly lower, and the relative membrane permeability of roots was significantly lower. This may be due to osmoprotective compounds can directly remove ROS or cause the protection of antioxidant enzymes (Chen et al., 2016). Exogenous H_2_S could reduce the degree of waterlogging damage to peach seedlings root systems, maintain the relative integrity of root cell membranes, reduce the damage from waterlogging to the root cell structure, ensure the normal physiological function of the root system, and maintain relatively high physiological activity.

Many studies have shown that H_2_S participates in the response to hypoxia stress in animal cells (Peng et al., 2010). Does H_2_S participate in the response to waterlogging stress in plant cells? Our results suggest that H_2_S may be involved in the response of plant cells to hypoxia stress. The results showed that waterlogging stress increased the content of endogenous H_2_S in root cells, and the application of exogenous H_2_S could increase the content of endogenous H_2_S in the root system. Toxic metabolites, such as ROS, methane, acetaldehyde, and phenols, accumulate in the cells under the stresses of waterlogging and hypoxia, eventually leading to plant poisoning. Under the conditions of hypoxia, due to the sharp decline or even lack of terminal electron receptor O_2_ levels, more ROS will be generated due to electron leakage than can be eliminated finally causing damage to cell structures, such as the cell membrane (Hamanaka and Chandel, 2009).

The results showed that exogenous H_2_S could significantly reduce the accumulation of ROS in peach leaves under waterlogging stress. In addition, studies have shown that H_2_S can also enhance the ability of plant cells to resist abiotic stresses, such as drought, by enhancing the activity of the antioxidant system (Jin et al., 2013; Li et al., 2013b, 2014; Lai et al., 2014). Combined with the measurement results of the endogenous H_2_S content in the root system of peach trees, this suggests that H_2_S can improve the activity of the antioxidant system of peach plants, reduce the accumulation of ROS in the cells, and finally effectively alleviate the damage caused by waterlogging and hypoxia stress. Pan et al. recently showed that H_2_S down regulated the expression of NADPH Oxidase 4 (Nox4) in mouse cardiomyocytes and ultimately reduced the content of ROS (Steffens et al., 2012). Chen et al. (2014) also showed that H_2_S significantly increased the content of GSH and decreased the accumulation of ROS. However, it is still controversial whether H_2_S can remove ROS. It reported that H_2_S increased ROS content in Arabidopsis root cells by regulating NADPH oxidase and glucose-6-phosphate dehydrogenase (G6PDH; Chen et al., 2014). Therefore, the effect of H_2_S on ROS metabolism needs further study.

Waterlogging can change the activity of antioxidant enzymes to different degrees (Ahmed et al., 2002; Yordanova et al., 2004). Improving the activity of antioxidant enzymes in plants under stress is very important concerning the ability to resist stress. The activities of antioxidant enzymes such as SOD and CAT in maize leaves were increased (Blokhina et al., 2000) under short-term waterlogging stress, and SOD and CAT in mungbean cells were increased at the early stages of waterlogging so as to eliminate the free radicals accumulated in cells (Ahmed et al., 2002). Our research shows that exogenous H_2_S can effectively improve the root protective enzyme activity under waterlogging stress, enhance the stress resistance of plants, and reduce the damage effect of stress on plants. In addition, our determination of O_2_^–^ and H_2_O_2_ content in root cells showed that waterlogging significantly increased the O_2_^–^ generation rate and H_2_O_2_ content in the root system of peach seedlings, while exogenous H_2_S could reduce the O_2_^–^ generation rate and H_2_O_2_ accumulation in the root system of peach seedlings under waterlogging stress (Figure 6). This may be due to the fact that exogenous H_2_S improves the activity of root protective enzymes under waterlogging stress, and the antioxidant enzymes, in plants can remove the active oxygen, reduce the accumulation of ROS in peach roots under waterlogging stress, and avoid cell injury (Blokhina et al., 2000; Ahmed et al., 2002; Ke et al., 2003), indicating that exogenous H_2_S is able to enhance the antioxidant capacity of peach seedlings. This enhancement may be the physiological basis for H_2_S-induced waterlogging stress tolerance in plants. The protective mechanism of H_2_S against oxidative damage was correlated with the enhanced activities of detoxifying enzymes under abiotic stress (Mostofa et al., 2015). The treatment of adding the H_2_S scavenger HT eliminated/partially eliminated the effect of H_2_S. The results showed that exogenous H_2_S could reduce the accumulation of ROS in the root system of peach seedlings under waterlogging stress, thereby alleviating the oxidative damage to the roots caused by waterlogging stress. In addition, waterlogging stress resulted in ethylene accumulation in submerged tissues. The mechanism stress tolerance mediated by ethylene has been gradually disclosed in recent years (Liu et al., 2011; An et al., 2018). Waterlogging stress caused the production of ethylene in rice roots, leading to increased synthesis of hydrogen peroxide, and eventually causing death of root cells (Steffens and Sauter, 2009). The H_2_S-mediated improved antioxidative system might have improved cell membrane integrity and might have repaired cell membrane injury by overcoming the oxidative damage (Ahmad et al., 2020). He et al. (1996) also showed that low oxygen treatment significantly increased ethylene content in roots. Exogenous H_2_S treatment increased the content of endogenous H_2_S, inhibiting the synthesis of ethylene in peach roots and reducing the damage from waterlogging stress. The mechanism of exogenous H_2_S reducing ethylene synthesis in peach roots under waterlogging stress remains to be further studied.

Chlorophyll is the material basis of photosynthesis in plant leaves, and its content can reflect the growth status and photosynthetic capacity of leaves (Nieva et al., 2005). The results show that the decreased oxygen supply in roots caused by waterlogging will lead to the closing of stomata and the decrease of stomatal conductance, thereby decreasing net photosynthetic rate, and the quantum efficiency of photochemistry (Ahmed et al., 2002; Carvalho and Amancio, 2002). At the same time, the decrease of total chlorophyll and chlorophyll a content in leaves will lead to decreases in plant growth and total biomass (Edwards et al., 2003). Chlorophyll fluorescence parameters reflect photosynthetic performance and the stress degree of plant leaves. PIABS is a parameter reflecting the comprehensive photosynthetic performance of plants. The fluorescence parameter Fv/Fm represents the primary light energy conversion efficiency of PS II (Demmig and Bjorkman, 1987). Studies have shown that low concentration of H_2_S can improve photosynthesis and regulate stomatal movement (Fang et al., 2014). On the one hand, it is believed that during the process of photosynthesis, H_2_S may increase the content of chlorophyll in plant leaves by changing the ultrastructure of chloroplasts so as to improve photosynthetic efficiency; on the other hand, this may be because H_2_S, as a signal molecule, enhances photosynthesis by regulating the activity of Rubisco, and the redox modification of mercapto compounds (Chen et al., 2011).

The accumulation of active oxygen and the decrease in photosynthetic rate are common responses of plants under waterlogging stress. The results showed that the chlorophyll content and net photosynthetic rate in young peach leaves was decreased under waterlogging stress, and the photosynthetic rate and Fv/Fm of leaves under exogenous H_2_S were significantly higher than those under waterlogging stress. In addition, compared with the non-waterlogging treatment, the stomatal openings of the flooded leaves were smaller, and the stomatal openings of the flooded leaves were significantly smaller than those of the control. In addition to terrestrial plants, in submerged macrophytes, H_2_S could rapidly induce biochemical responses, photosynthesis, and plant growth, which further adapts to aquatic environment (Parveen et al., 2017). Combined with the data for active oxygen metabolism in leaves, exogenous H_2_S appeared to significantly reduce the O_2_^–^ production rate and H_2_O_2_ content of peach leaves under waterlogging stress, improve the photosynthetic rate, and alleviate stress damage by regulating the antioxidant metabolism of plants. H_2_S might act as an antioxidant to inhibit or scavenge ROS productions for maintaining the lower MDA and H_2_O_2_ levels, and thereby preventing oxidative damages (Chen et al., 2017). The results showed that exogenous H_2_S alleviated the damage to the photosynthetic mechanism and maintained relatively high net photosynthetic rate and electron efficiency.

**FIGURE 9:**
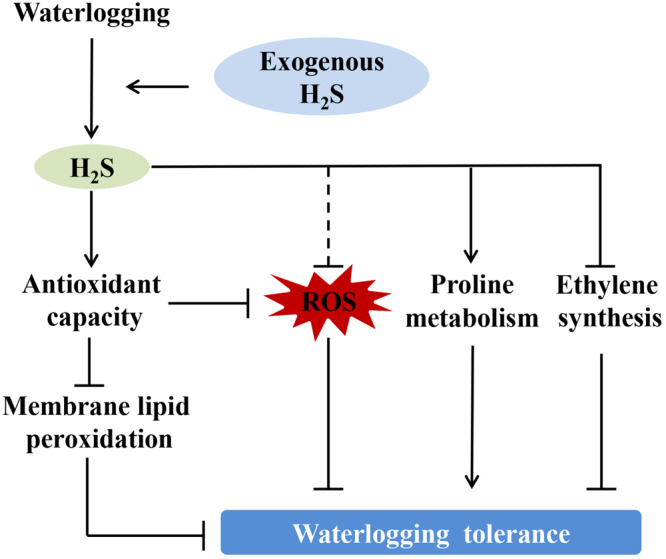
Schematic illustration for possible mechanism of hydrogen sulfide improving peach seedlings’ waterlogging tolerance. H_2_S: Hydrogen sulfide, ROS: Reactive oxygen species.

A schematic illustration for a possible mechanism of H_2_S improving peach seedlings’ waterlogging tolerance is presented in Figure 9. Our results suggest that under waterlogging stress, exogenous application of H_2_S increased the stomatal opening, chlorophyll content, and photosynthetic capacity of peach seedlings leaves and activated the ROS scavenging system, thereby reducing oxidative damage. Exogenous H_2_S could significantly alleviate hypoxia-induced cell membrane damage by enhancing the ROS scavenging system and inhibiting ethylene synthesis in peach seedlings. H_2_S treatment improved the seedlings’ photosynthetic capacity and chlorophyll content, thereby enhancing the tolerance of the plants to hypoxic stress. The results suggest that H_2_S treatment can improve fruit trees’ waterlogging tolerance. This is particularly significant, because waterlogging stress has become an ecological crisis for fruit tree production, especially for peach trees with poor hypoxic stress tolerance. However, further research is needed to investigate the physiological and molecular signaling mechanisms through which exogenous H_2_S increases the waterlogging tolerance of peach seedlings.

## Data Availability Statement

All datasets generated for this study are included in the article/supplementary material.

## Author Contributions

FP and YX conceived and designed the experiments. XW, MS, and YX performed the experiments. XW and MS contributed to reagents, materials, and analysis tools. YX, XW, and FP wrote the manuscript.

## Conflict of Interest

The authors declare that the research was conducted in the absence of any commercial or financial relationships that could be construed as a potential conflict of interest.
